# Dead-end complex, lipid interactions and catalytic mechanism of microsomal glutathione transferase 1, an electron crystallography and mutagenesis investigation

**DOI:** 10.1038/s41598-017-07912-3

**Published:** 2017-08-11

**Authors:** Qie Kuang, Pasi Purhonen, Johan Ålander, Richard Svensson, Veronika Hoogland, Jens Winerdal, Linda Spahiu, Astrid Ottosson-Wadlund, Caroline Jegerschöld, Ralf Morgenstern, Hans Hebert

**Affiliations:** 1 Department of Biosciences and Nutrition, Karolinska Institutet and School of Technology and Health, Royal Institute of Technology, SE-141 83 Huddinge, Sweden; 2 Institute of Environmental Medicine, Karolinska Institutet, SE-171 77 Stockholm, Sweden

## Abstract

Microsomal glutathione transferase 1 (MGST1) is a detoxification enzyme belonging to the Membrane Associated Proteins in Eicosanoid and Glutathione Metabolism (MAPEG) superfamily. Here we have used electron crystallography of two-dimensional crystals in order to determine an atomic model of rat MGST1 in a lipid environment. The model comprises 123 of the 155 amino acid residues, two structured phospholipid molecules, two aliphatic chains and one glutathione (GSH) molecule. The functional unit is a homotrimer centered on the crystallographic three-fold axes of the unit cell. The GSH substrate binds in an extended conformation at the interface between two subunits of the trimer supported by new *in vitro* mutagenesis data. Mutation of Arginine 130 to alanine resulted in complete loss of activity consistent with a role for Arginine 130 in stabilizing the strongly nucleophilic GSH thiolate required for catalysis. Based on the new model and an electron diffraction data set from crystals soaked with trinitrobenzene, that forms a dead-end Meisenheimer complex with GSH, a difference map was calculated. The map reveals side chain movements opening a cavity that defines the second substrate site.

## Introduction

Glutathione (GSH) is a γ−L-Glu-L-Cys-Gly tripeptide with a gamma peptide linkage between the amine group of cysteine and the carboxyl group of the glutamate side-chain. As a potent physiological reducing agent, GSH is the most abundant intracellular small molecule thiol, reaching millimolar concentrations in most cell types in higher organisms^1^. GSH plays a key role in redox regulation and the detoxification of a variety of electrophilic compounds and peroxides via catalysis by glutathione transferases (GST)^2^, glutathione peroxidases (GPx)^3^ and peroxiredoxins^4^.

GSTs constitute one of the most important groups of phase II detoxification enzymes. They are abundantly expressed throughout most life forms. GSTs catalyze the conjugation of GSH to a wide variety of endogenous and exogenous electrophilic compounds^5^ with hydrophobic character. Based on subcellular localization and structure, GSTs can be divided into the membrane bound microsomal/mitochondrial as well as soluble cytosolic and mitochondrial family members^6^. The integral polytopic membrane inserted GSTs are distinctly different from the soluble ones^7^. While the functional unit is a homotrimer in membranes, the dominant catalytically active organization of soluble GSTs is a dimer. It was observed that the most well studied membrane bound GST, microsomal glutathione transferase 1 (MGST1, EC number: 2.5.1.18) shared similarities with leukotriene C4 synthase (LTC4S, EC number: 4.4.1.20), catalyzing the conjugation reaction between leukotriene (LT) A4 and GSH. Recently, characterization of MGST2 has revealed similarities also to this enzyme regarding substrate specificity and third-of-the-sites-reaction mechanism^8, 9^. It is now well established that MGST1, MGST2 and LTC4S belong to the membrane associated proteins in eicosanoid and glutathione metabolism (MAPEG) superfamily^7^. In humans also MGST3 is a member of MAPEG, together with microsomal prostaglandin E synthase 1 (MPGES1) and 5-lipoxygenase activating protein (FLAP). The MGSTs and LTC4S catalyze conjugation reactions to GSH. While the structure of LTC4S defines this protein’s specificity for the LTA4 substrate^10, 11^, MGST1 is less exclusive; a property that is compatible with its function as a detoxification enzyme^12^. MPGES1 primarily catalyzes the GSH dependent isomerization of prostaglandin (PG) H2 to PGE2, but a low GST and GPx activity has also been demonstrated^13^. Among the six human members of MAPEG, FLAP is the only one for which no catalytic function has been detected. Instead FLAP is involved in leukotriene production by interacting with 5-lipoxygenase by an as yet undefined mechanism. A common theme of MAPEG proteins is their interaction with, and catalytic conversion of, endogenous reactive lipids in signaling or protection from lipid peroxidation.

Here we have studied the molecular structure of a GST in membrane bound form: MGST1. As for all GSTs, MGST1 has the capacity to lower the pKa value of the sulfhydryl group of bound GSH^14^. The enzyme stabilizes the thiolate anion at neutral pH and combines this capability with providing a binding pocket for hydrophobic electrophiles at a site adjacent to the bound GSH^15^. Apart from this important role in phase II detoxification, MGST1 has been shown also to have GSH peroxidase activity against lipid hydroperoxides^16^. The localization of MGST1 to membranes thus suggested that this enzyme is important for protection against lipid peroxidation, as was demonstrated^16^. From studies in *Drosophila* it has been suggested that MGST1 activity may be linked to aging^17^. By disrupting a MGST1 like gene it was demonstrated that the mutant flies had a significantly reduced life-span as compared to controls. Since many clinical useful cytostatic drugs are also potential substrates for GSTs, development of drug resistance e.g. in cancer treatment, can be an important factor leading to treatment failure. MGST1 has^18, 19^, like the cytosolic GSTs, been linked with the development of resistance toward chemotherapy agents^20, 21^ in humans and also pesticides^22^ in insects.

In order to shed light on the catalytic pathway of MGST1, analysis of the structural properties of MGST1 were investigated early on. We prepared two-dimensional (2D) crystals and eventually determined an atomic model of the protein with bound GSH^15^. The enzyme was purified from rat liver. The structure was based on phase and amplitude Fourier component information from cryo electron microscopy (cryoEM) images and electron diffraction, respectively. Already from projection maps we concluded that MGST1 assembles into a trimer in the membrane^23^. Furthermore, the 3D model resolved the topology of the monomer as a four-helix bundle and placed the active site at the interface between monomers in the trimer^15^. X-ray structures of LTC4S^10, 11^ and MPGES1^24^, with better completeness of experimental data and more revealing contribution of high resolution information, confirmed these observations and could map further details with regard to binding of GSH.

Three outstanding questions that need to be answered are: is the GSH conformation in MGST1 really different from that in other members of the MAPEG superfamily, can we get information on the access pathway of the lipophilic second substrates and is there a residue essential for catalysis (i.e. lowering the pKa of the GSH thiol in the active site) as has been shown for other MAPEGs? For the latter question, we present specific mutations of MGST1 influencing catalysis. In particular, Arginine 130 is here shown to be essential for catalysis in analogy with other MAPEGs.

In order to enable more extensive comparisons of structures within the MAPEG superfamily, including the opportunity to address possible influences of the surrounding environment (phospholipid vs detergent), we have now determined a refined structure of MGST1. It is based on electron diffraction recordings from 2D crystals of the p6 type. Instead of continuing with enzyme purified from rat liver we now used protein heterologously expressed in *Escherichia coli* (*E. coli*). The number of diffraction patterns was increased from 100 to 225. The new model comprises 123 of the 155 amino acid residues, two structured phospholipid molecules, two hydrocarbon chains and one GSH molecule. Interactions between subunits form trimers centered on the crystallographic three-fold axes of the unit cell. Also in our refined structure, the GSH substrate binds in an extended conformation at the interface between two subunits of the trimer.

We have also collected an electron diffraction data set from 2D crystals of MGST1 containing GSH following soaking with 1,3,5-trinitrobenzene (TNB). In the presence of this molecule it is known that the catalytic process is stalled at the formation of a Meisenheimer dead-end complex^25^ enabling studies of an otherwise transient intermediate. Structural differences were observed based on amplitude differences between the Fourier components of the diffraction data sets yielding information on the second substrate access path and chemical mechanism.

## Results

### Extended conformation of glutathione and mutations in the active site

**Figure 1:**
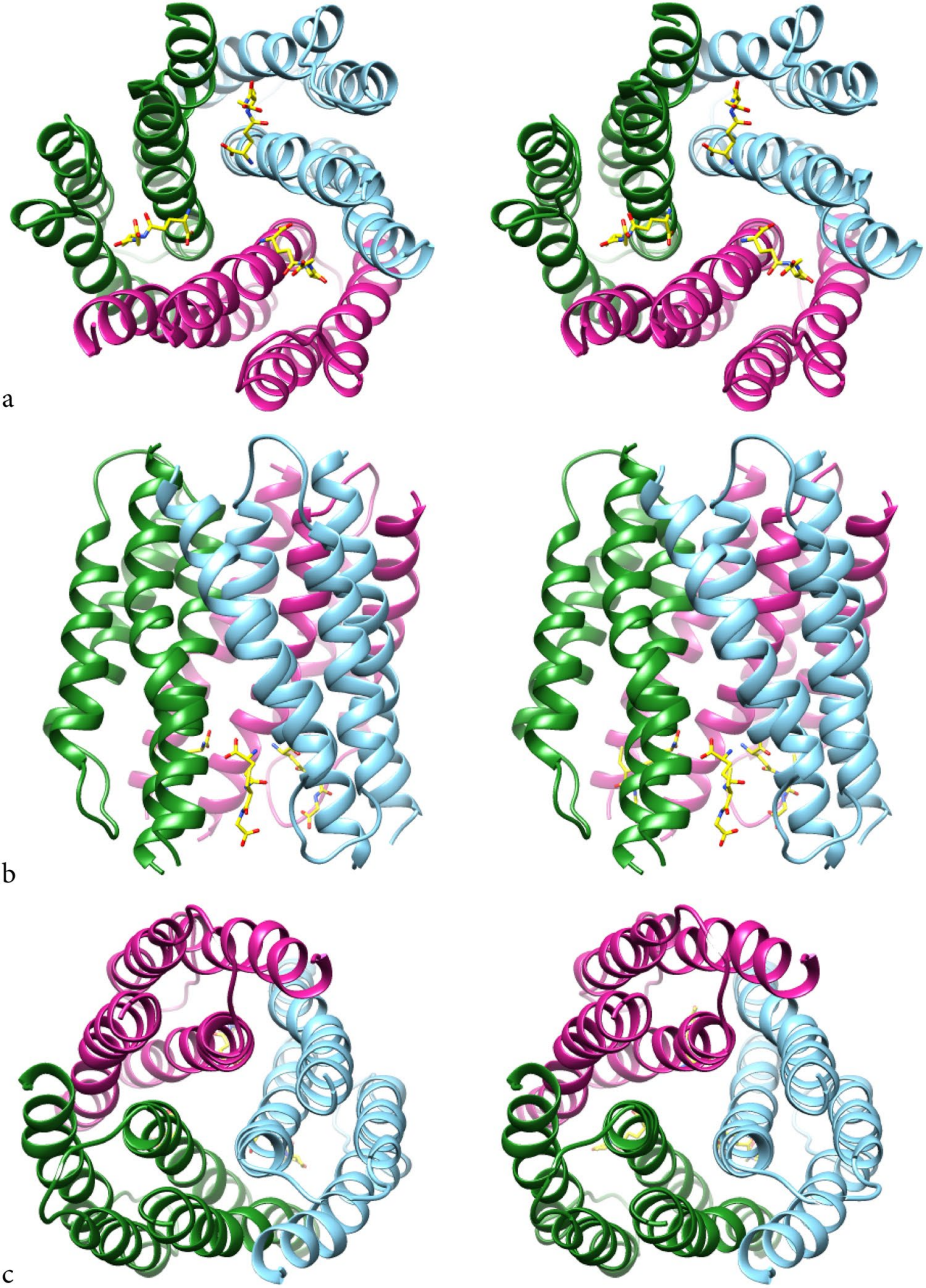
Helical packing of MGST1. Cross-eyed stereo views of the functional trimer with GSH in yellow. (**a**) Cytoplasmic view. (**b**) side view with cytoplasmic side facing down. (**c**) ER lumenal top view. GSH is located between subunits coloured differently.

MGST1 contains 155 amino acids out of which 123 could be included in the model (Fig. 1, for the sequence, see Supplementary Fig. S1). The four transmembrane (TM) helices and two loops were sufficiently ordered to contribute to density that could be interpreted in terms of an atomic model whereas the connection between TM helices 1 and 2 was not (Supplementary Fig. S2). Residues 43 to 65 were therefore not included in the model. As was pointed out by Sjögren *et al*.^24^ for the corresponding loop in MPGES1 (denoted C-loop) a direct amino acid interaction to the GSH from this loop was not observed in the MPGES1 X-ray structure (PDB ID: 4AL0) and can neither be found in the MPGES1 structure obtained by 2D-crystallography (PDB ID: 3DWW)^26^. As the residue identity between MPGES1 and MGST1 is partly high for the TM1-TM2 loop (in this cytoplasmic loop, 5 out of 8 residues among K42-D49 are conserved between MGST1 and MPGES1), it may be the case also for MGST1 that this loop is not directly involved in GSH binding but serves other purposes.

**Figure 2:**
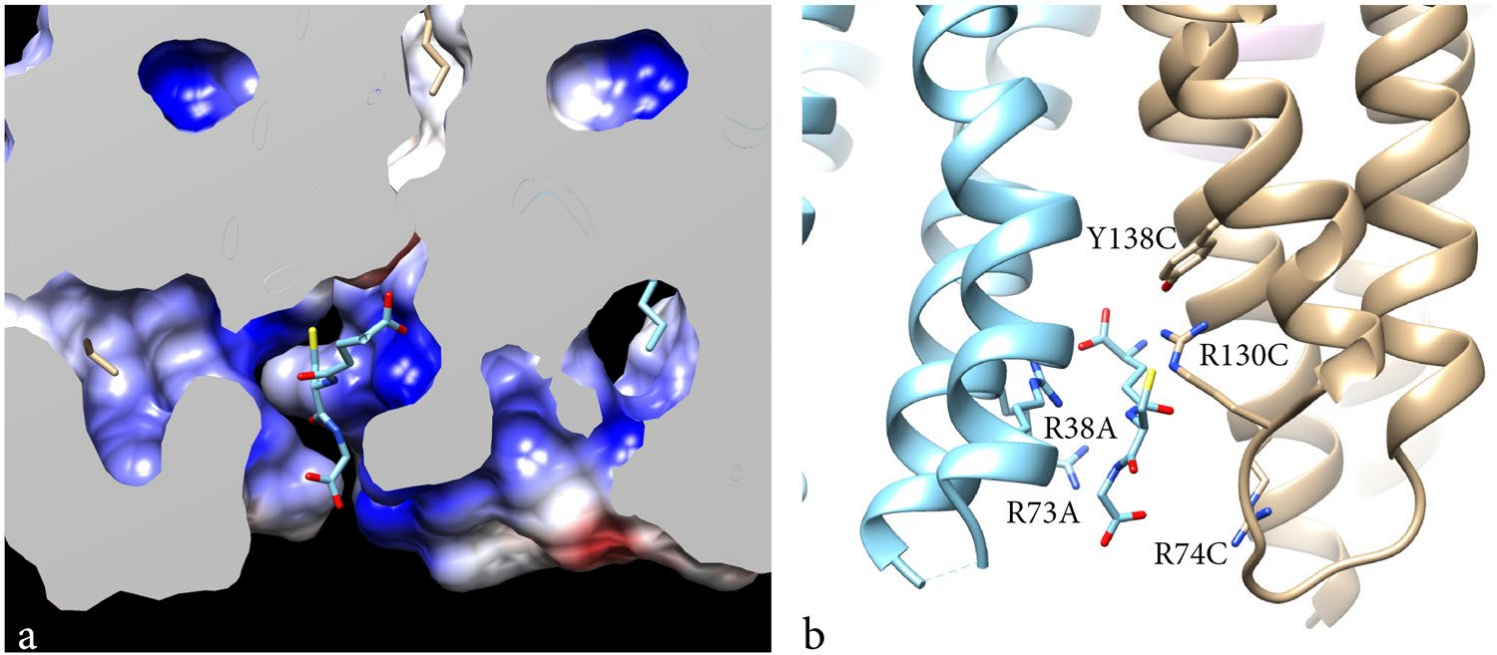
Shape and binding of GSH in MGST1. (**a**) Binding pocket for GSH and (**b**) residues in the vicinity of GSH. Residues R38 and R73 from one subunit (suffix A) and R130 and Y138 from a neighbour subunit (suffix C) in the trimer are in close contact with the same GSH molecule.

The functional unit of MGST1 is a homotrimer (Fig. 1). A difference map calculated between observed (with GSH) and model (without GSH) amplitudes and using model phases had the strongest density at a position facing the cytoplasmic side and at the interface between adjacent monomers of the MGST1 trimer (Supplementary Fig. S3). These densities were interpreted to arise from bound GSH molecules (Figs 1 and 2). The positions are similar to those observed for MGST1^15^, LTC4S (PDB ID: 2UUH and 2PNO) and MPGES1 (PDB ID: 3DWW and 4AL0). In contrast, the shape of the density and subsequent modelling suggested that GSH is bound in an extended conformation (Fig. 2). Trials to fit a horseshoe shaped molecule, as has been observed both in LTC4S (PDB ID: 2UUH and 2PNO) and MPGES1 (PDB ID: 3DWW and 4AL0), were not successful and led to reduction of refinement statistical criteria. In the new structure, residues close to GSH are: A35, R38, L39, K42, R73, H76, L77, L80 from one subunit and R74, L77, N78, E81, Q127, N129, R130 from its neighbour.

A new feature in our model is the hydrogen bond between the cysteine thiol of GSH and R130 (Fig. 2b). Mutation of this residue to alanine resulted in complete loss of activity consistent with a role for Arginine 130 in stabilizing the strongly nucleophilic GSH thiolate required for catalysis (Table 1). The position of GSH in the model is supported by data showing that mutation of two of the arginines, R73 and R74, results in complete loss of substrate saturation with GSH (with CDNB as the electrophilic second substrate). The mutant H76Q, which is close to the active site, increases the K_m_ for GSH, measurably 6–7 fold. Below, we include an extensive discussion of published and new *in vitro* mutagenesis data that support the assigned location of GSH.

**Table 1:** Specific activity and kinetic parameters of MGST1 mutants expressed in *E. coli.*

Mutation	Specific activity (µmol min^−1^ mg^−1^)	K_m_ GSH (mM) (0.5 mM CDNB)	K_m_ GSH (mM) (0.5 mM CNAP)
Wt	6.4 ± 0.58	7.5 ± 0.4	2.1 ± 0.85^e^
R38A	5.5 ± 1.5		
R73A	3.7 ± 1.2^c^	NS^b^	33 ± 9.4
R74Q	2.4 ± 0.51^c^	NS	220 ± 43
H76Q	1.2 ± 0.16^d^	45 ± 11	23 ± 3.8
N78T	ND^a,c^		
E81Q	0.51 ± 0.061^c^		
R130A	ND^a^		
Y138F	13^d^		

^a^Not detectable, ^b^Not saturable, ^c^[ref. 57], ^d^[ref. 27], ^e^[ref. 58].

### Structural differences in the presence of an inhibitor: 1,3,5-trinitrobenzene

**Figure 3:**
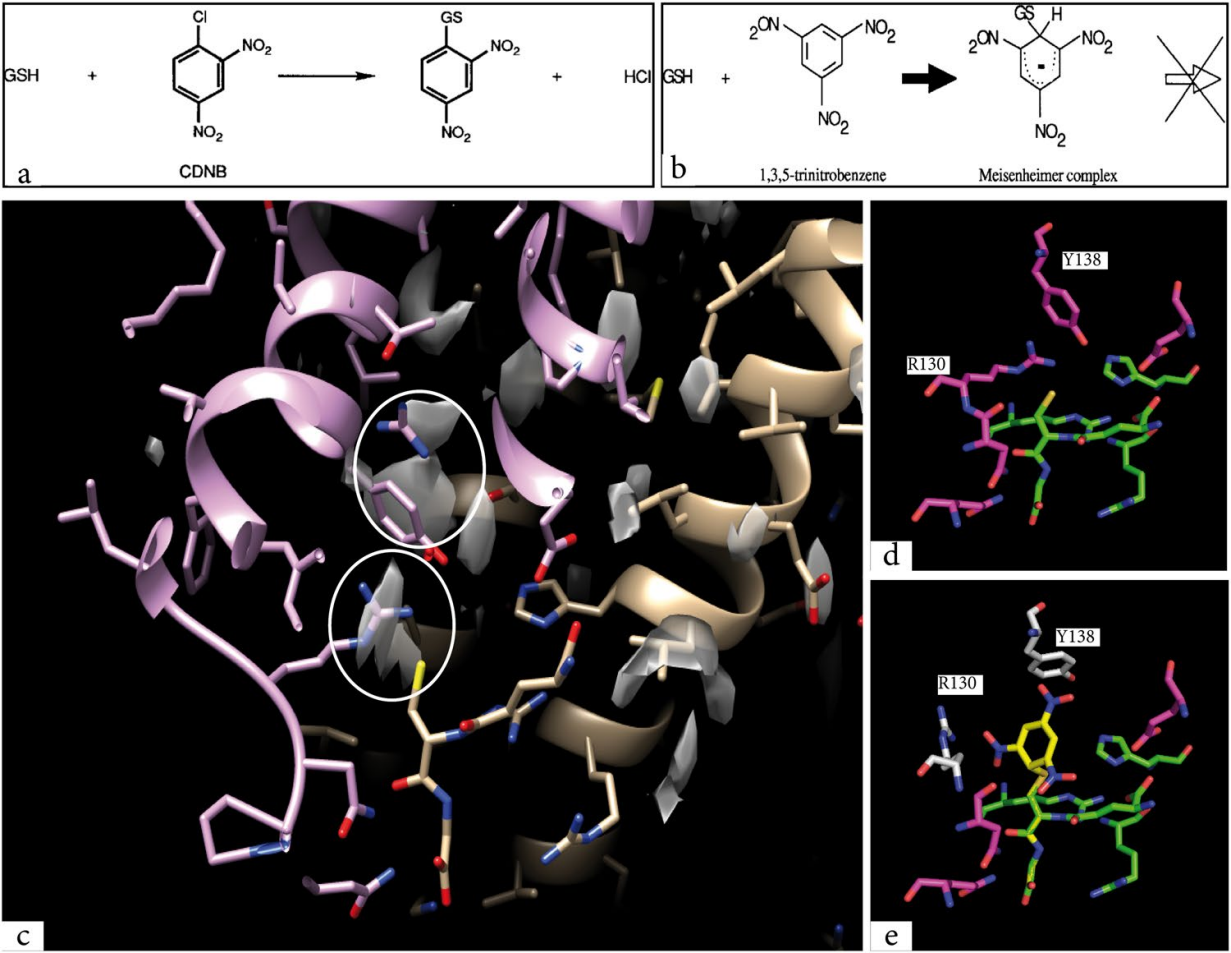
Formation of the reaction intermediate, the Meisenheimer complex. (**a**) Glutathione transferases act on lipophilic electrophiles bound to their hydrophobic binding site, positioning the substrate in close proximity to the activated thiol in GSH. (**b**)1,3,5-Trinitrobenzene (TNB), which lacks a good leaving group, is known to reversibly form a Meisenheimer complex. (**c**) Difference map between TNB and native data shows peaks in the difference map (σ = 2.6) close to the GSH molecule. (**d**) Side chain positions in the native model. (**e**) Tyr 138 side chain movement to match the upper difference-peak as shown in (**c**) and position of the TNB moiety to match the lower peak. Putative position of Arg130 to open the binding site for TNB.

In the presence of TNB as the second substrate, the catalysis is stalled after the conjugation of TNB on GSH (Fig. 3a and b). The dead-end complex formed between GSH and the bulky TNB remains bound in the active site allowing for a structural investigation. 2D crystals were soaked with TNB and electron diffraction patterns recorded for a 3D data set (Supplementary Table S4, TNB data set; Supplementary Figs S5 and S6 and Supplementary methods). A difference map was calculated using the amplitudes from the two data sets and phases from the present MGST1 model. The strongest difference peaks appeared close to the position of the GSH and residue Y138 on TM4 (Fig. 3c and Supplementary Fig. S7) while no significant changes were observed at other positions in the unit cell close to the atomic model. Mutation of tyrosine 138 to a phenylalanine resulted in a doubling of the specific activity (Table 1) indicating a specific role for this tyrosine^27^ as a gatekeeper and moderator of second substrate access.

### Lipid location and overall structure details

**Figure 4:**
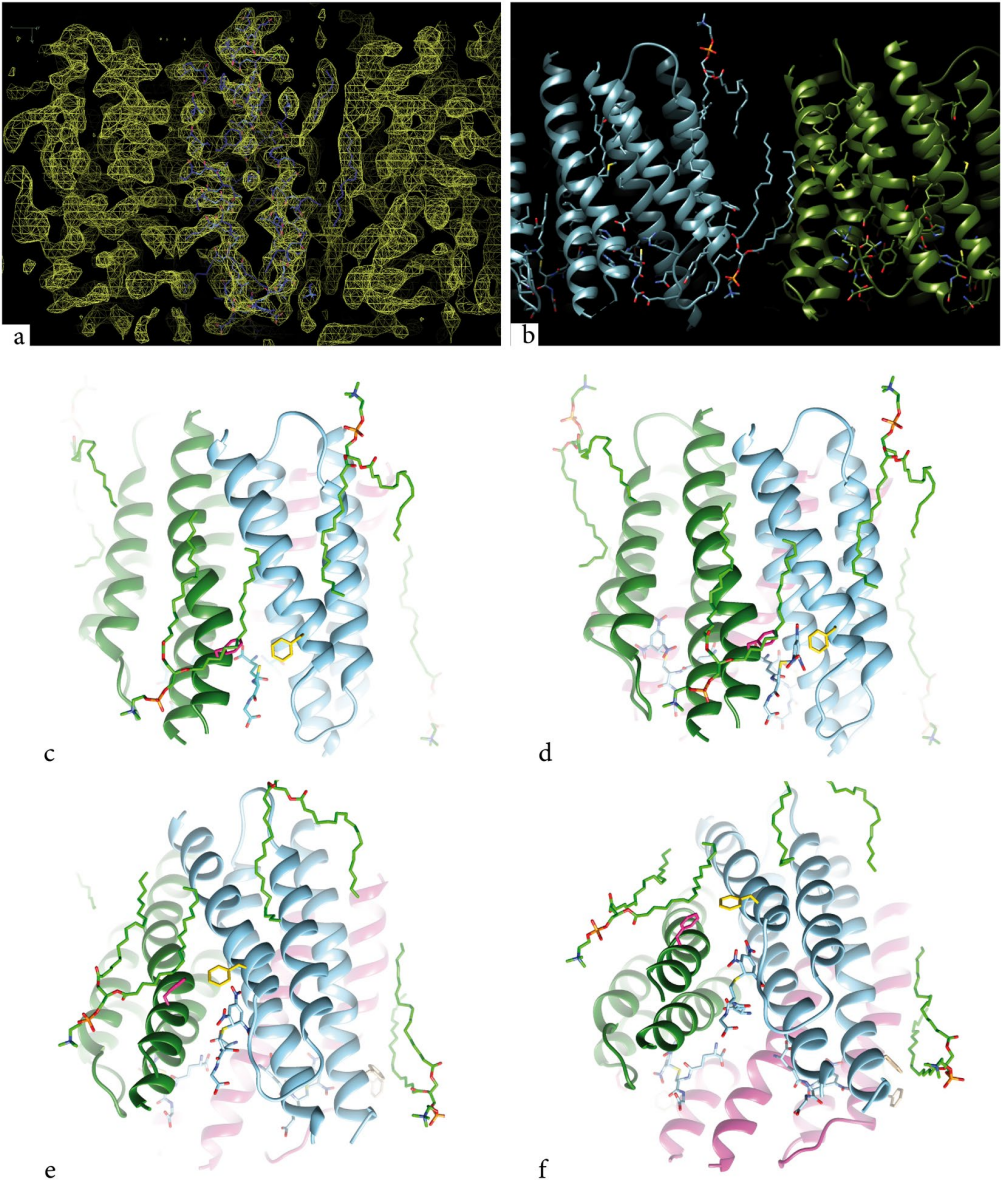
Two specific lipids in MGST1, one on the luminal side and one on the cytosolic side. The latter covers the putative second substrate entry path. (**a**) Side view of the 2Fo-Fc map at 1.2 σ with transmembrane helices 3 and 4 in the foreground. Parts of the two modelled phospholipids can be seen to the right of the helical domains. (**b**) Side view of two trimers of MGST1 with two phospholipid molecules at the interface. (**c**) Side view of MGST1 showing the two lipids (bright green) and GSH (cyan). F36 in magenta and F135 in yellow. (**d**) Side view of MGST1 showing the dead-end complex of GSH-TNB (light blue). (**e**) and (**f**) increasingly tilted views of MGST1 indicating that the entry path is rather closed (**e**) and the active site well within the protein trimer (**f**).

MGST1 molecules form two trimers centered on the three-fold axes of the p6 unit cell (Supplementary Fig. S8). At the interface between those two trimers, close to the two-fold axis, two independently ordered phospholipid molecules could be resolved (here modelled as di-stearoyl-3-sn-phosphatidylcholine) facing opposite sides of the membrane (Fig. 4). Figure 4a and b show the lipids to occupy specific, well defined volumes between trimers. The side view of MGST1 (Fig. 4c) shows that one lipid (with the head group facing the cytosol) occupies most of the area outside the cleft between TM1 and TM4 of different subunits. One of the acyl chains covers phenylalanine F36 on TM1. This residue and F135 on TM2 are shown as they are located where the substrate entry from the membrane is expected. The other lipid, with the head group facing the lumen, covers a region near the clefts between TM1-TM3 and TM3-TM4 (Fig. 4c).

As compared to the previous model of MGST1^15^, built from weak side chain densities, there are frame shifts with regard to the new model for TM3 and TM4. Moreover, the C-terminal end of TM1 now continues further towards the cytoplasmic side. The present model was also compared to the human LTC4S structure (PDB ID: 2UUH) in Supplementary Fig. S9a. The sequence alignment of rat MGST1, human MGST1, human LTC4S and human MPGES1 using the residue numbering specified for rat MGST1, including the N-terminal methionine, is shown in Supplementary Fig. S1. The root-mean-square deviations (rmsd in Å) after optimized fit of subsets of Cα atoms in MGST1 and LTC4S were: TM1 (residues 10–42) 1.66, TM2 (65–95) 1.518, TM3 (104–120) 0.713, TM4 (133–155) 1.452 with an overall value of 2.42. Deviations varied along the helical stretches with largest rmsd-values towards both ends of TM1 and the luminal side of TM2. Analogous to MPGES1 and opposite to FLAP, the MGST1 structure shows a cone shaped cavity open to the cytoplasmic side of the ER whereas the luminal side shows no opening. This is due to differences in the position of a proline in TM2 (Supplementary Fig. S1).

Several specific contacts were identified within each monomer of MGST1. An ion-pair, K26/D79, located in the lipid bilayer region connects TM1 and TM2 (Supplementary Fig. S9b). Aromatic-aromatic interactions from closely positioned phenylalanine side chains are formed between TM2 and TM3 (Supplementary Fig. S9c). A hydrogen bonding network forms a tight interaction in the center of the MGST1 monomer (Supplementary Fig. S9d). Intermolecular contacts between monomers in the trimer are formed between H76 and E81 from two adjacent TM2s (Supplementary Fig. S9e). As mentioned above, mutation of the latter residues leads to a pronounced lowering of the MGST1 activity and mutation of the former leads to an activity decrease as well as a 6–7 fold increase in K_m_ for GSH (Table 1).

## Discussion

The glutathione conformation observed here in MGST1 is extended (Figs 1 and 2). Furthermore, the location of the GSH density in the present study is clearly supported by mutagenesis data of residues suggested to contact GSH (Table 1). Functional studies have shown that MGST1 has one-third-of-the-sites-reactivity^28^, which may be coupled to conformational differences in the trimer and/or a combination of catalytically competent and non-competent binding modes for GSH. In spite of this, the GSH density peak had sufficiently high signal to noise ratio and shape for modelling the tripeptide in an extended conformation. Other MAPEG members, LTC4S and MPGES1, have been found to bind GSH in a unique horseshoe shape^10, 11, 24, 26^ and it has been suggested that MGST1 could also exhibit this binding conformation^29^. However, two arguments support a different GSH binding mode in MGST1: 1) in electron crystallography maps of MPGES1 the horseshoe shape of GSH could be observed at a similar resolution^26^, and 2) MGST1 has the unique ability to use N-acetyl-L-cysteine as substrate supporting a different GSH site (discussed further below). The extended conformation of GSH in the MGST1 structure is more similar to what has been found in soluble glutathione binding proteins including GSTs^30^. As MGST1 displays third-of-the-sites-reactivity the possibility remains that extended and horseshoe conformations can co-exist at different active sites in the trimer or transiently during catalysis.

It may seem surprising that so many residues in the GSH binding site can be mutated leaving the activity largely preserved. However, a more detailed analysis of the rate behavior upon varying GSH revealed striking effects (Table 1). The enzyme can no longer be saturated with GSH in the R73 and R74 mutants leading to the paradoxical situation that mutation decreases activity at low GSH but increases activity at very high GSH concentrations. R74 is conserved in all MAPEG with a catalytic GSH dependent activity whereas R73 is conserved in MGST1 only. Similarly a structural alteration in the second sphere GSH binding region (H76Q) resulted in a marked increase of the K_m_ for GSH. Mutation of certain proximal residues did result in loss of activity (N78 and R130 discussed below). There is additional support for a unique character of the MGST1 GSH interaction. Several of the GSH interacting residues (R73R74-H76-N78-(I/L80)E81) are present in a conserved peptide sequence pattern that constitutes a specific diagnostic for MGST1 in phylogeny^31^.

Earlier we studied the GSH binding requirements of MGST1 using GSH analogues. We found that several analogues were substrates including N-acetyl-L-cysteine^14^. This analogue displays a very high K_m_ and at high concentrations the activity with the analogue becomes comparable and even higher than that with GSH. From these observations we can conclude that the MGST1 GSH binding site appears to be rather malleable. This appears to be unique to MGST1 as soluble GSTs and MPGES1 do not accept N-acetyl-L-cysteine as substrate. Hence MGST1 displays unique structural elements and functional properties consistent with a different GSH binding conformation as compared to other MAPEG members.

MGST2 and LTC4S display a sequence identity as high as 44%. Both catalyse the conjugation of GSH to leukotriene A4. Whereas LTC4S shows an all sites reactivity^32^, MGST2 shows a 1/3 sites reactivity^33^ akin to MGST1. The loop between TM1 and TM2 forms a lid over the active site in LTC4S and would have to fold away to allow for an extended conformation of GSH. The residue identity is high and the number of residues in the loop is identical in LTC4S and MGST2 pointing to GSH being shaped as a horseshoe also in MGST2. Thus, the extended conformation of GSH might not to be a prerequisite for the 1/3 sites reactivity.

Nevertheless 1/3 of the sites reactivity implies conformational heterogeneity in MGST1. Our structure appears to capture a catalytically competent state where the GSH thiol is in proximity to an arginine, located in TM4, essential for catalysis.

A crucial step following GSH-binding in many GSH-dependent enzymes is stabilization of the thiolate anion at physiological pH^34^. For most GSTs the pK_a_ of the GSH thiol is lowered from 9^35–37^ to a value between 6 and 7^14^. In soluble GSTs belonging to the classes alpha, mu, pi, and sigma, the thiolate anion is being stabilized by hydrogen bonding with a tyrosine residue and by interactions with hydrating water molecules^34^ whereas in other GSTs a serine is utilized^38^. In LTC4S and MGST2 it has been shown that this role is taken by an arginine located in TM4 close to the loop connecting this helix to TM3^10, 11, 39^. This arginine is conserved in the MAPEG superfamily except in FLAP, which does not bind GSH and lacks catalytic activity. In the present MGST1 structure this arginine corresponds to R130 (Fig. 2b) within hydrogen bonding distance of the GSH sulphur. Mutation of this residue resulted in an inactive protein (Table 1) consistent with a role in thiolate anion stabilization. Regarding MPGES1, the closest relative to MGST1 at 38% sequence identity, structural data from a high resolution structure^24^ revealed that serine-127 was in close contact with the GSH thiol. Consequently, the involvement of the R126 (corresponding to R130 in MGST1) was questioned. However, mutation of S127 in MPGES1 did not affect activity^40^. A dynamic Asp–Arg interaction is essential for catalysis in MPGES1 where mutation of R126 results in loss of PGE synthase activity^40, 41^. Supporting the lack of a role for S127 in MAPEG catalysis, the corresponding position in MGST1 is occupied by glycine or alanine (rat and human enzyme, respectively). In conclusion, specific arginines remain the strongest candidates for GSH thiolate stabilization in enzymatically active MAPEGs: MGST1 (R130); MPGES1 (R126)^40, 42^; LTC4S (R104)^39^ and MPGES2 (R104)^32^. In MGST3 the candidate to be investigated is R114.

We have observed that soaking 2D crystals of MGST1 with TNB results in a Meisenheimer complex absorption spectra and also demonstrated that the enzyme is catalytically active in the lipid/protein 2D crystals. Formation of the MGST1/Meisenheimer complex does not induce major conformational changes. However, as observed in the difference maps, a strong local negative/positive peak pair was observed in the vicinity of the GSH and could be interpreted as a movement of the Y138 side chain (Fig. 3, Supplementary Fig. S7). An additional strong positive peak appeared at a position expected to be occupied by the TNB moiety of the dead-end complex. Additional movements of the side chain of Arg 130 would be required to open the binding pocket for TNB. In consequence, it is suggested that the concerted movement of the side chains of Y138 and R130 is an essential step in the catalytic mechanism of MGST1 leading to accommodation and stabilization of a Meisenheimer complex in the case of activated chloroaryl compounds.

Regarding the chemical mechanism, the role of GSH thiolate anion stabilization has been well documented for most GSTs^6^. Another aspect of GST catalysis by necessity involves transfer of a negative charge en route to, and as part of, the transition state (as the nucleophilic GSH thiolate attacks the electrophilic substrate). We suggest that R130 plays an important role in stabilization of this transition state. We base this suggestion on the modelled position of Arg 130 in the MGST1-TNB-Meisenheimer complex, suitable for stabilizing the delocalized negative charge of the charge transfer complex.

Like for bacterial rhodopsin (PDB ID: 2AT9 and 2BRD)^43, 44^ and aquaporin 0 (PDB ID: 2B6O)^45^ we could now model ordered phospholipids into the structure. For the MGST1 2D crystals with p6 two-sided plane group symmetry, two phospholipid molecules were found at the interface between subunits of adjacent trimers of the protein (Fig. 4a and b, Supplementary Fig. S8). One of these has the phosphate of the head group facing the luminal membrane orientation in the vicinity of the loop between TM2 and TM3 (Fig. 4c). However, no specific charge-neutralizing side chain could be identified. Threonine 102, in a few species replaced by a serine, is the closest residue with around 6 Å from the threonine oxygen to either an oxygen on the phosphate or to a carboxyl oxygen on one of the attached fatty acids. Also on the cytoplasmic facing side no specific interaction was evident between the protein and the phosphate of the second lipid localized close to the loop between TM3 and TM4. On the cytoplasmic side, Threonine 40, conserved in MGST1, was the closest residue with 4.4 Å from the threonine oxygen to one of the fatty acid carboxyl oxygens.

The acyl chains of the phospholipids fill up parts of the hydrophobic region between the two adjacent trimers. Interestingly, the cytosol-facing lipid occupies an area right outside TM1 and TM4 of different subunits, i.e. just outside the substrate entry area (Fig. 4c). F36 and F135 in TM1 and TM4 respectively are on 4.4 Å distance effectively closing the entry from the lipid bilayer. One acyl chain appears to cover the two phenylalanines keeping it closed. It is peculiar that the volume closed off behind the phenylalanines and lipid can house the TNB-GSH complex with such small changes in the organization of the active site (Fig. 4f). On the other hand, compared to MPGES1, the location of the cysteinyl sulfur is closer to the trimer center in MGST1. Possibly the extended and vertical shape in MGST1 allows the conjugation of second substrate within the enzyme in contrast to MPGES1 where most of the substrate appears to remain outside the protein in the lipid bilayer (PDB IDs 4AL1, 4YL0, 5BQH, 5BQG, 5K0I).

The locations of the defined phospholipids are consistent with the width of biological membranes and show that MAPEG enzymes are deeply buried with active site access for hydrophobic second substrates at the headgroup hydrocarbon interphase region. The position of the Meisenheimer complex can be taken as an indication of the lipophilic second substrate entry path (Fig. 4d–f) and, judging from the position of bound phospholipid, would occur close to the lipid headgroup-hydrocarbon chain interphase. This is a region where typical lipophilic substrates (that always contain some hydrogen bonding capacity) tend to accumulate^46^.

In summary, we have characterized a refined structural model of MGST1 revealing a unique GSH conformation (compared to other MAPEG), catalytically important residues, reaction intermediate stabilization and the entry path for lipophilic substrates via the membrane.

## Materials and Methods

### Recombinant expression in *E. coli*

The procedure of heterologous expression and purification of native rat MGST1 was described previously^47, 48^. Now, we expressed the recombinant rat MGST1 containing a six-histidine-tag at the N-terminus in C43 (DE3) strain (Lucigen), which gives a higher yield than BL21 (DE3) pLys SL strain used previously. The culture in Terrific Broth medium was grown until OD_600_ = 0.25 at 37 °C and then the temperature was reduced to 30 °C for 20 minutes. At this time point, the cells were induced with 0.5 mM IPTG and followed by incubation overnight.

### Purification

The purification procedure is similar as in^47^ with the following modifications: 1) The membrane fraction was solubilized by addition of an equal volume of: 10 mM sodium phosphate (NaP_i_) at pH 8, 10% glycerol, 0.1 mM EDTA, 1 mM GSH, and 6% Triton X-100 (Sigma) and followed by 45 minutes incubation in 4 °C; 2) The hydroxyapatite affinity chromatography was replaced by immobilized metal ion affinity chromatography (IMAC), (column: Hitrap chelating, from GE healthcare). The unspecific proteins were washed in 10 mM NaP_i_ at pH 8, 150 mM NaCl, 1 mM GSH, 10% glycerol, 50 mM imidazole, and 0.1% reduced Triton X-100 (Sigma) and rat MGST1 protein was eluted in 350 mM imidazole; 3) After IMAC, the eluted peak was immediately desalted (column: HiPrep 26/10, from GE healthcare) in buffer A: 10 mM NaP_i_ at pH 8, 30 mM NaCl, 1 mM GSH, 10% glycerol, and 0.1% reduced Triton X-100. The desalted sample was further purified by cation exchange chromatography (column: Hitrap SP, from GE healthcare) equilibrated with buffer A. Rat MGST1 protein was eluted with 300 mM NaCl in buffer A. The pooled fractions (approximate 6 ml) were concentrated (approximate 0.6 ml) (centrifugal tube: Amicon Ultra-4 centrifugal filter unit with 10 kDa cutoff, from Millipore) and followed by 2D crystallization trials. The activity measurement was performed according to^49^.

### Mutant generation

The N-terminal 6xHis version of plasmid pSP19T7LTMGST1, containing wild-type rat MGST1 was used as the template for site-directed mutagenesis^47^. Forward primers for mutagenesis were:

R38A: CTC CGC GAC TGC ATT CCA GGC GCT AAC CAA CAA GGT TTT TG

R130A: CCC TTC CTC AGC CAA ACG CGG GCT TGG CAT TTT TTG

Mutants were expressed and activity measured in membrane fractions as described^47^. Expression was verified and quantitated by Western blot^47^.

### 2D crystallization

The crystallization procedure was performed as described^15^ with a different crystallization buffer: 25 mM Tris-HCl at pH 7.4, 20% glycerol, 0.1 mM EDTA, 100 mM KCl, 1 mM GSH, and 50 mM CaCl_2_ and crystallization was performed at 30 °C.

Formation of the Meisenheimer complex was made by mixing 0.3 μl of saturated TNB solution in ethanol with 2.5 μl of MGST1 crystal suspension, which contains 1 mM GSH. For a single EM grid prepared as described below it normally took 5 min from mixing to freezing. Initially we also incubated suspensions of crystals with TNB for 30 minutes prior to grid preparation but this did not improve diffraction. Stopped-flow measurements had also shown that formation of the complex is almost instantaneous^50^.

### Electron diffraction recording

The 2D crystals, both native and incubated with TNB, were embedded in trehalose and prepared for cryoEM using the inverted grid technique^51^ prior to freezing in liquid nitrogen and transferring to a JEOL 2100 F electron microscope (JEOL). Electron diffraction patterns (Supplementary Fig. S5) were recorded on a 4k × 4k CCD camera equipped with a software-controlled retractable beam stopper (TVIPS Video and Image Processing Systems, Gauting, Germany). Exposure times were 5–20 s and the stage goniometer was set at nominal tilt angles between 0° and 60°.

### Data processing

Selected diffraction patterns were processed with programs from the MRC suite^52^ in order to perform indexing and retrieve integrated, background corrected intensities. LATLINE was used to obtain equidistant samples along the adapted lattice line curves (Supplementary Fig. S6). Subsequent processing was performed with programs from CCP4^53^. Refmac5^54^ was used for refinement and Coot^55^ for model building. Figures were made from Pymol (The PyMOL Molecular Graphics System, Version 1.3r1.edu Schrödinger, LLC) and Chimera^56^.

Coordinates and structure factors have been deposited in the Protein Data Bank with accession numbers 5I9K and 5IA9 for the MGST1/GSH and the MGST1/Meisenheimer structures, respectively. Corresponding 2Fo-Fc maps have been deposited in the Electron Microscopy Data Bank with ID codes EMD-8076 and EMD-8084.

## Electronic supplementary material

Supplementary Information
