# Supplementary material for: Dead-end complex, lipid interactions and catalytic mechanism of microsomal glutathione transferase 1, an electron crystallography and mutagenesis investigation
Source: Sci Rep. 2017 Aug 11;7:7897. doi: 10.1038/s41598-017-07912-3 (PMC5554250; doi:10.1038/s41598-017-07912-3)
Supplement: Supplementary file 1 — Supplementary Information [file 41598_2017_7912_MOESM1_ESM.pdf]

Supplementary Information

Dead-end complex, lipid interactions and catalytic mechanism of microsomal glutathione transferase 1, an electron crystallography and mutagenesis investigation

Qie Kuang[1], Pasi Purhonen[1], Johan Ålander[2], Richard Svensson[2], Veronika Hoogland[2], Jens Winerdal[2], Linda Spahiu[2], Astrid Ottosson-Wadlund[2], Caroline Jegerschöld[1], Ralf Morgenstern[2] and Hans Hebert[1]

[1]Department of Biosciences and Nutrition, Karolinska Institutet and School of Technology and Health, Royal Institute of Technology, SE-141 83 Huddinge, Sweden

[2]Department of Environmental Medicine, Karolinska Institutet, SE-171 77 Stockholm, Sweden

```
modelid           10         20         30         40         50         60
rMGST1  MADLKQLMDNEVLMAFTSYATIILAKMMFLSSATAFQRLTNKVFANPEDCAGFGKGENAKKF
hMGST1  MVDLTQVMDDEVFMAFASYATIILSKMMLMSTATAFYRLTRKVFANPEDCVAFGKGENAKKY
hLTC4S  --------MKDEVALLAAVTLLGVLLQAYFSLQVISARRAFRVSPPL---------------
hMPGES1 MPAHSLVMSSPALPAFLLCSTLLVIKMYVVAIITGQVRLRKKAFANPEDALRHG----GPQY


modelid         70         80         90         100        110        120
rMGST1  LRTDEKVERVRRAHLNDLENIVPFLGIGLLYSLSGPDLSTALIHFRIFVGARIYHTIAYLT
hMGST1  LRTDDRVERVRRAHLNDLENIIPFLGIGLLYSLSGPDPSTAILHFRLFVGARIYHTIAYLT
hLTC4S  TTGPPEFERVYRAQVNCSEYFPLFLATLWVAGIFFH-EGAAALCGLVYLFARLRYFQGYAR
hMPGES1 CRSDPDVERCLRAHRNDMETIYPFLFLGFVYSFLGPNPFVAWMHFLVFLVGRVAHTVAYLG


modelid        130        140        150        160        170
rMGST1  PLPQPNRGLAFFVGYGVTLSMAYRLLRSRLYL----------------------------
hMGST1  PLPQPNRALSFFVGYGVTLSMAYRLLKSKLYL----------------------------
hLTC4S  SA--QLRLAPLYASARALWLLVALAALGLLAHFLPAALRAALLGRLRTLLPWA--------
hMPGES1 KLRAPIRSVTYTLAQLPCASMALQILWEAARHL---------------------------
```

Supplementary Fig. S1 Sequence alignment with Clustal W between rat and human microsomal glutathione transferase 1 (rMGST1 and hMGST1), human lekotriene C4 synthase (hLTC4S) and human microsomal prostaglandin E2 synthase 1 (hMPGES1). Numbering, as used in this paper starts at the MGST1 N-terminal methionine residues and follows those sequences without gaps.

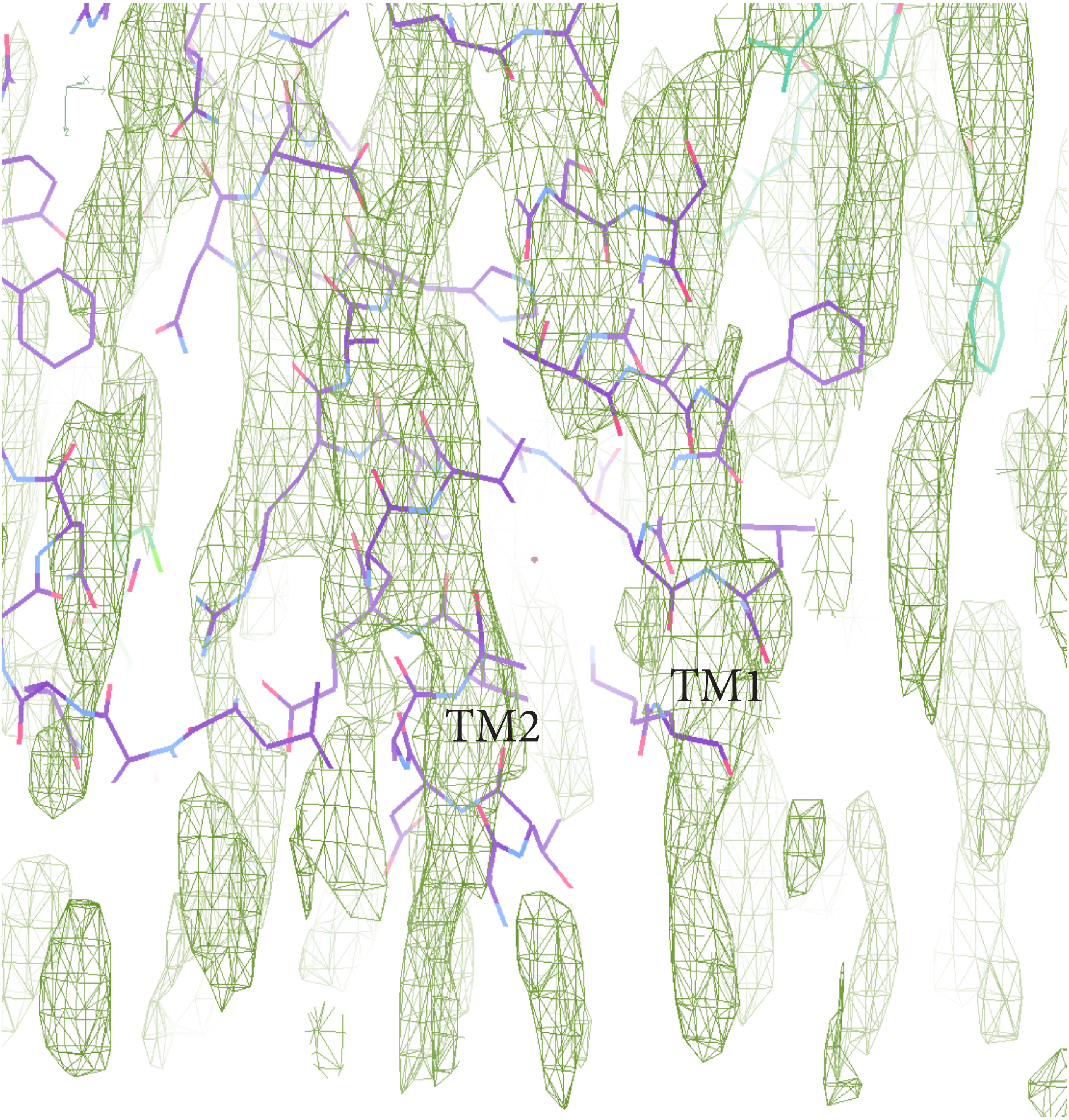

Supplementary Fig. S2 Raw map calculated from electron diffraction pattern amplitudes collected in this work and phase information from previous image data (ref 15 in the main text) at the expected position of the connection between TM1 and TM2. No temperature factor was applied. The overlay shows the present model of MGST1.

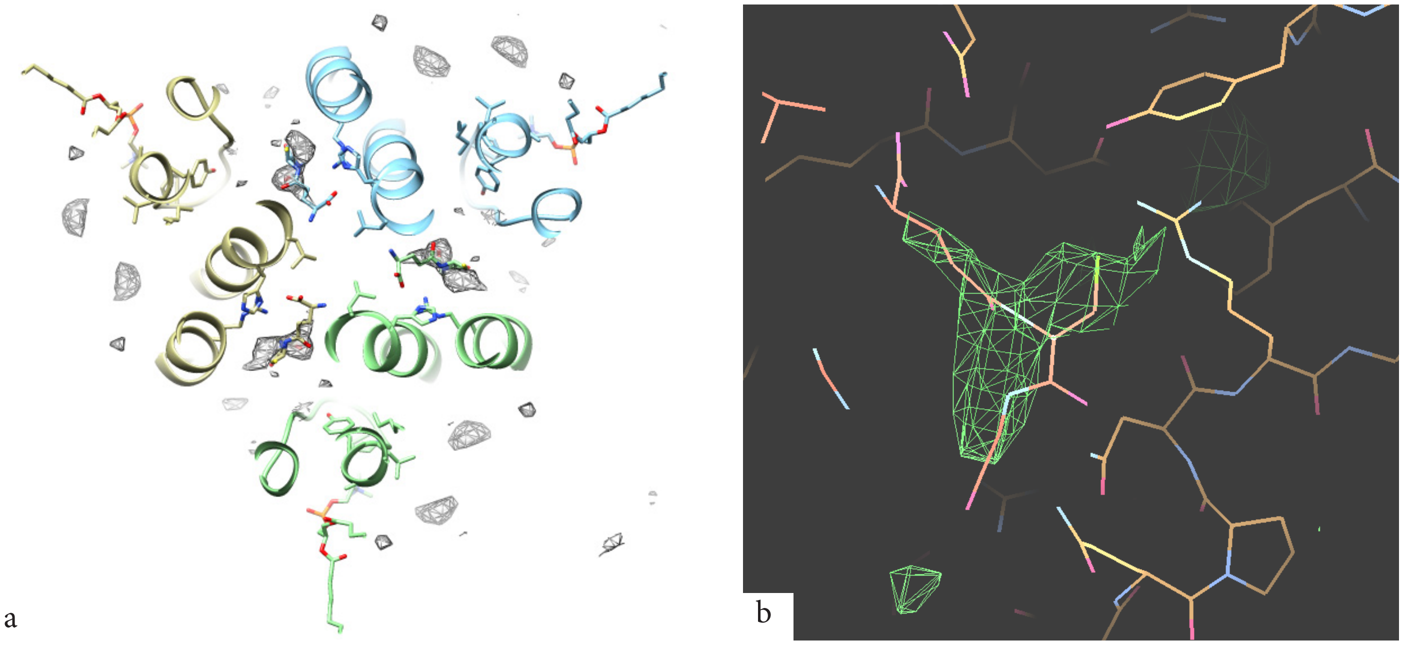

Supplementary Fig. S3 (a) Localization and conformation of glutathione in the MGST1 trimer. The Fo-Fc map is depicted at 3σ threshold. (b) Close-up of the Fo-Fc map and model of GSH. The Fo-Fc map is depicted at 3σ threshold.

Supplementary Table S4. Crystal parameters, data collection and refinement statistics

	Native data	TNB data
**Two-dimensional crystal parameters**		
Two-sided plane group	p6	p6
Unit cell dimension		
a (Å)	81.8	81.8
b (Å)	81.8	81.8
c (Å)[a]	100.0	100.0
γ (°)	120.0	120.0
**Electron diffraction data collection**		
No of diffraction patterns	225	86
Maximum tilt angle (°)	66.3	57.4
Resolution in and normal to membrane plane (Å)[b]	3.0/4.0	3.0/4.0
No of observed/used amplitudes	97987/6314	43603/5539
Fourier space sampled, overall/3.5 – 3.0 Å (%)	82.5/72.3	72.4/62.8
F/σ, overall/3.5 – 3.0 Å	7.66/4.60	6.57/4.19
B-factor (from Wilson plot)	43.6	56.3
$R_{Friedel}$ (%)	12.6	12.0
R_{merge} (%)	39.6	34.3
**Crystallographic refinement**		
Resolution	10.0 – 3.5 Å	10.0 – 3.5 Å
No. of reflections	3799	3063
No. of atoms protein/substrate/phospholipids	984/20/128	984/35/128
R_{work} (%)	27.0	28.2
R_{free} (%)	29.3	30.0
r.m.s deviations		
Bond lengths	0.025	0.026
Bond angles	3.12	3.17
Ramachandran plot distribution (%)	80.7/16.8/2.5	80.7/16.8/2.5

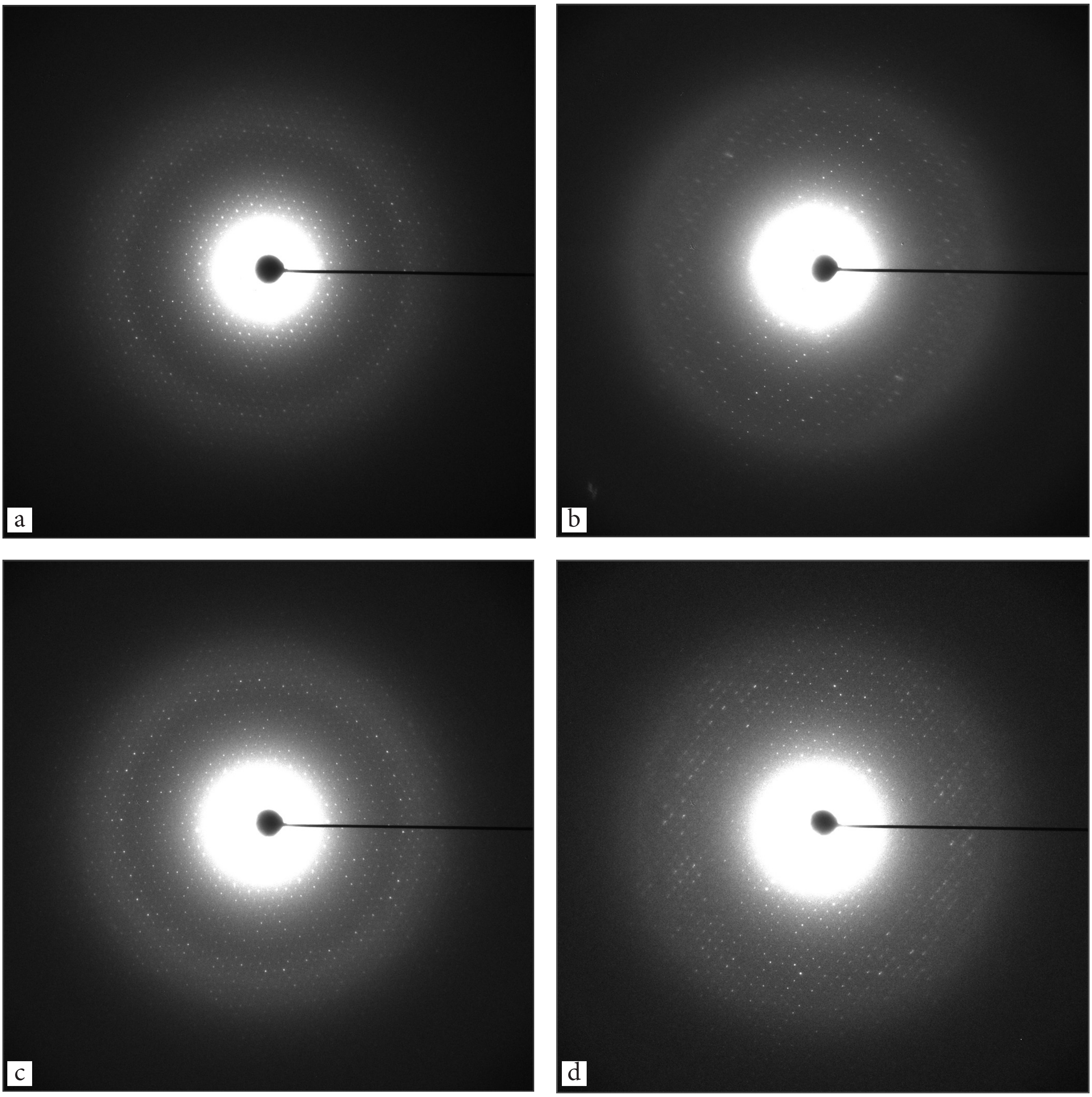

Supplementary Fig. S5 Electron diffraction patterns from 2D crystals of MGST1. Native crystal at tilt angles 0° (a) and 56° (b). Crystals soaked with TNB at tilt angles 0° (c) and 45° (d). The size of the p6 unit cell is a=b=81.8 Å.

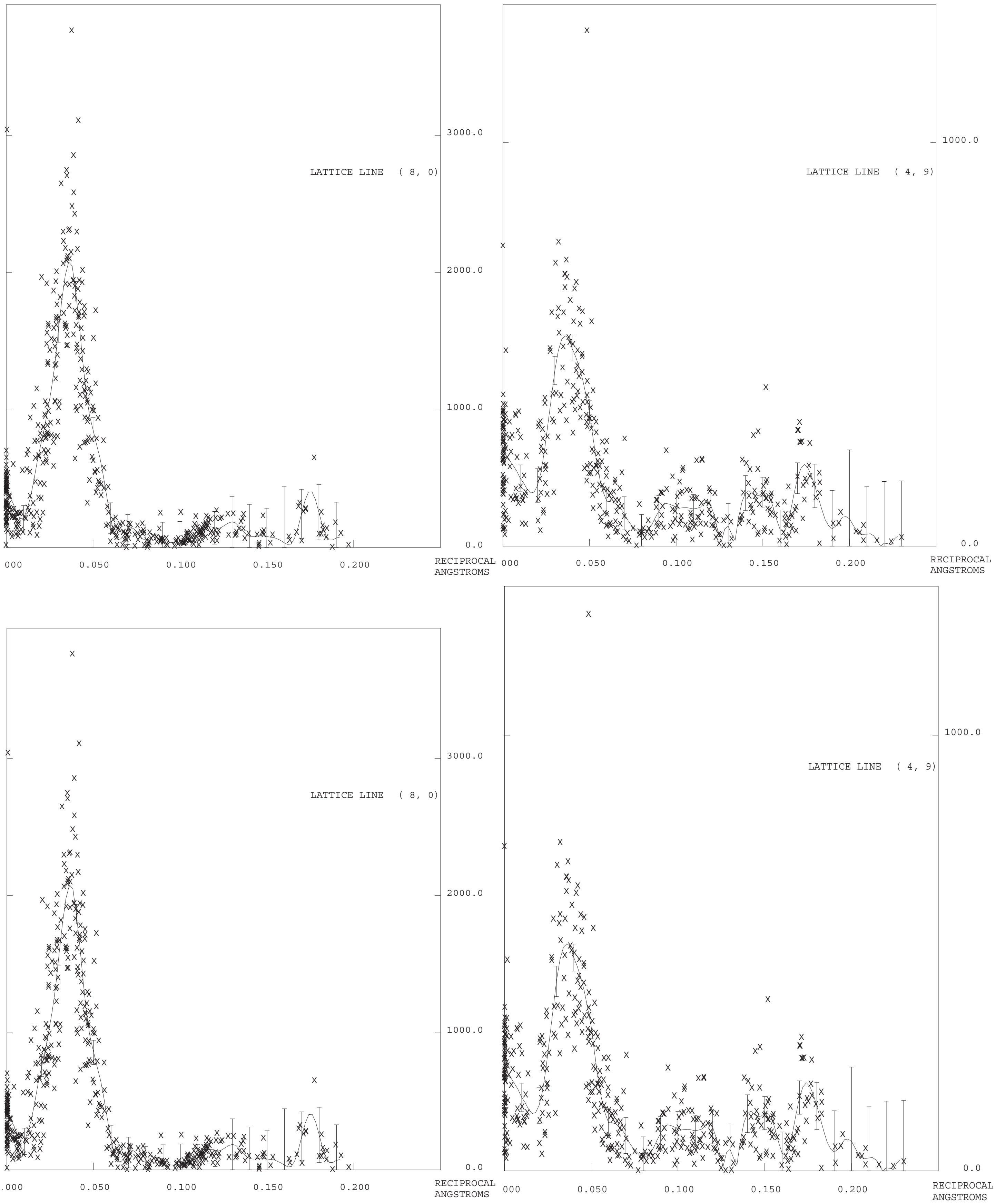

Supplementary Fig. S6 Measured diffraction intiensities and adapted curves along lattice lines (8.0) and (4,9) for the native (upper) and TNB (lower) data sets.

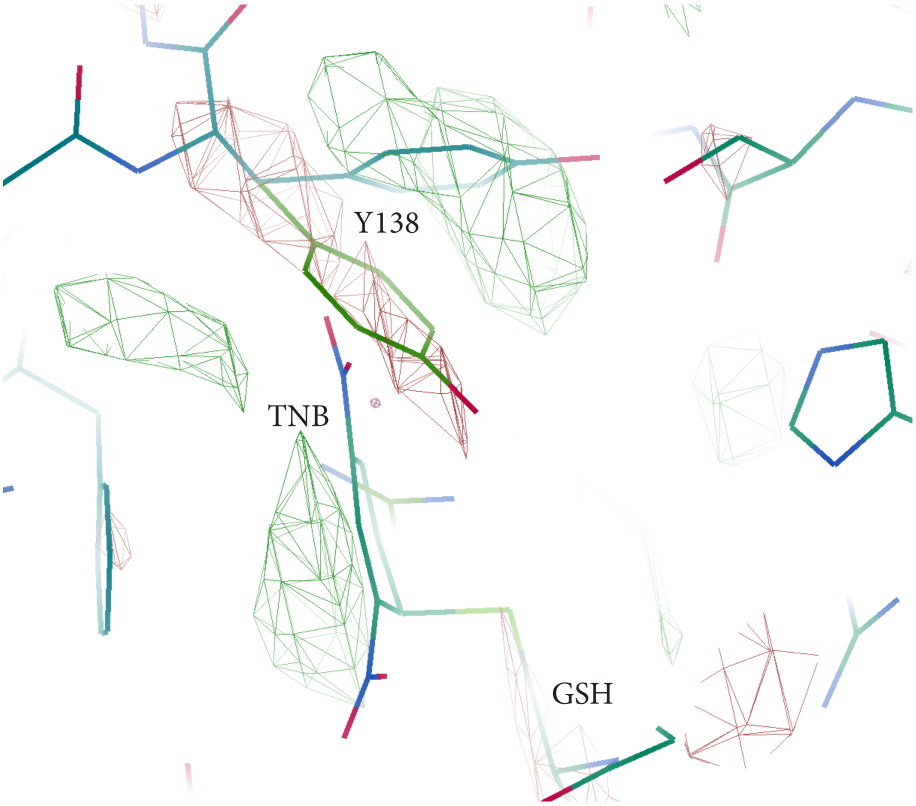

Supplementary Fig. S7 Peaks in the difference map calculated between amplitudes from the native MGST1/GSH and the MGST1/Mesienheimer complex data sets using the phases from the MGST1/GSH model. Positive (green) and negative (red) peaks are shown at σ=3.0.

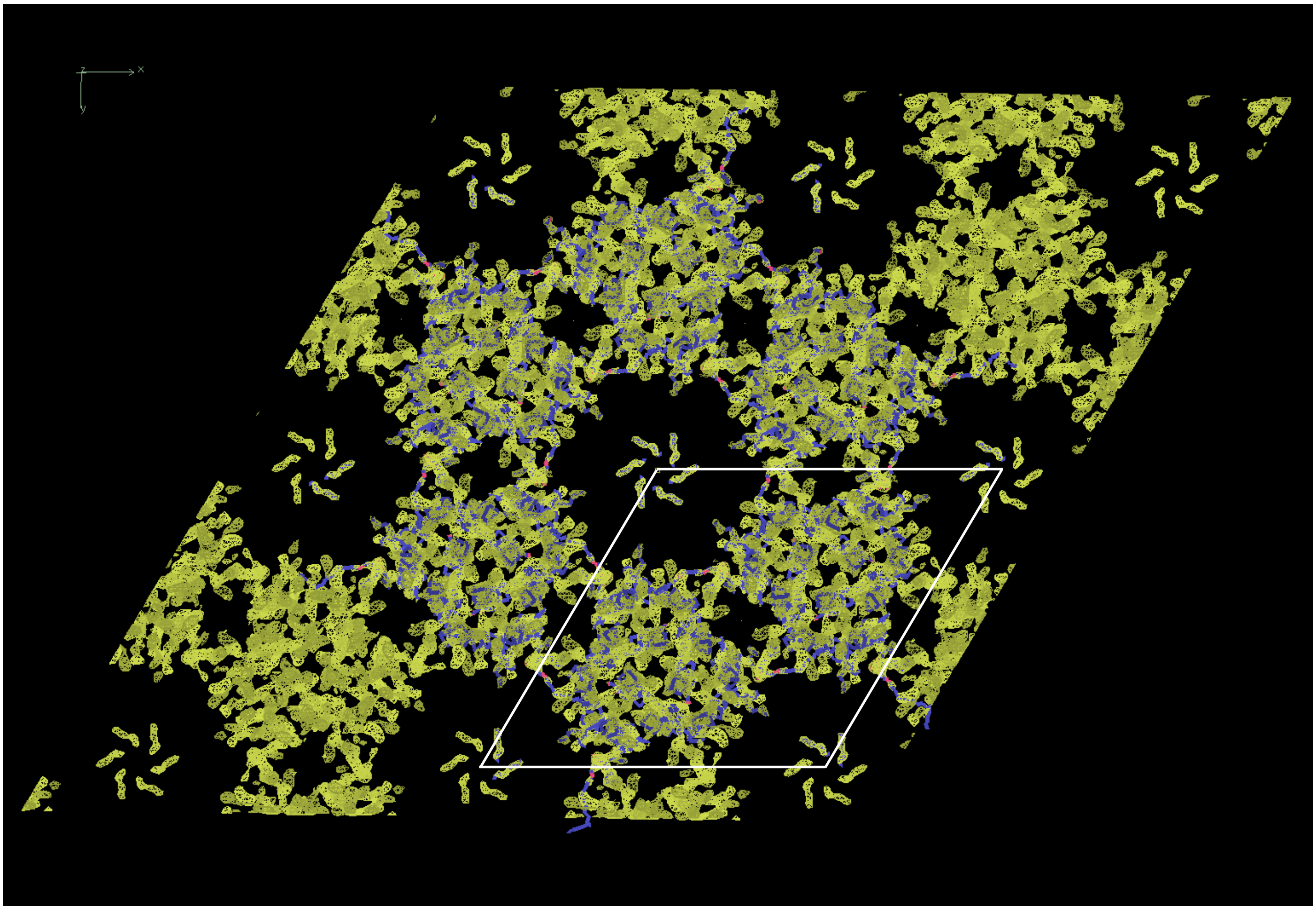

Supplementary Fig. S8 Packing of MGST1 in 2D crystals. The outline of the p6 unit cell with a=b=81.8 Å as viewed along the c-direction perpendicular to the membrane plane. An initial model is shown in blue. It contains two phospholipid molecules per monomer at the interface between the two trimers in the unit cell. The depicted 2Fo-Fc map was contoured at a high threshold value (2.0 σ) to increase visibility.

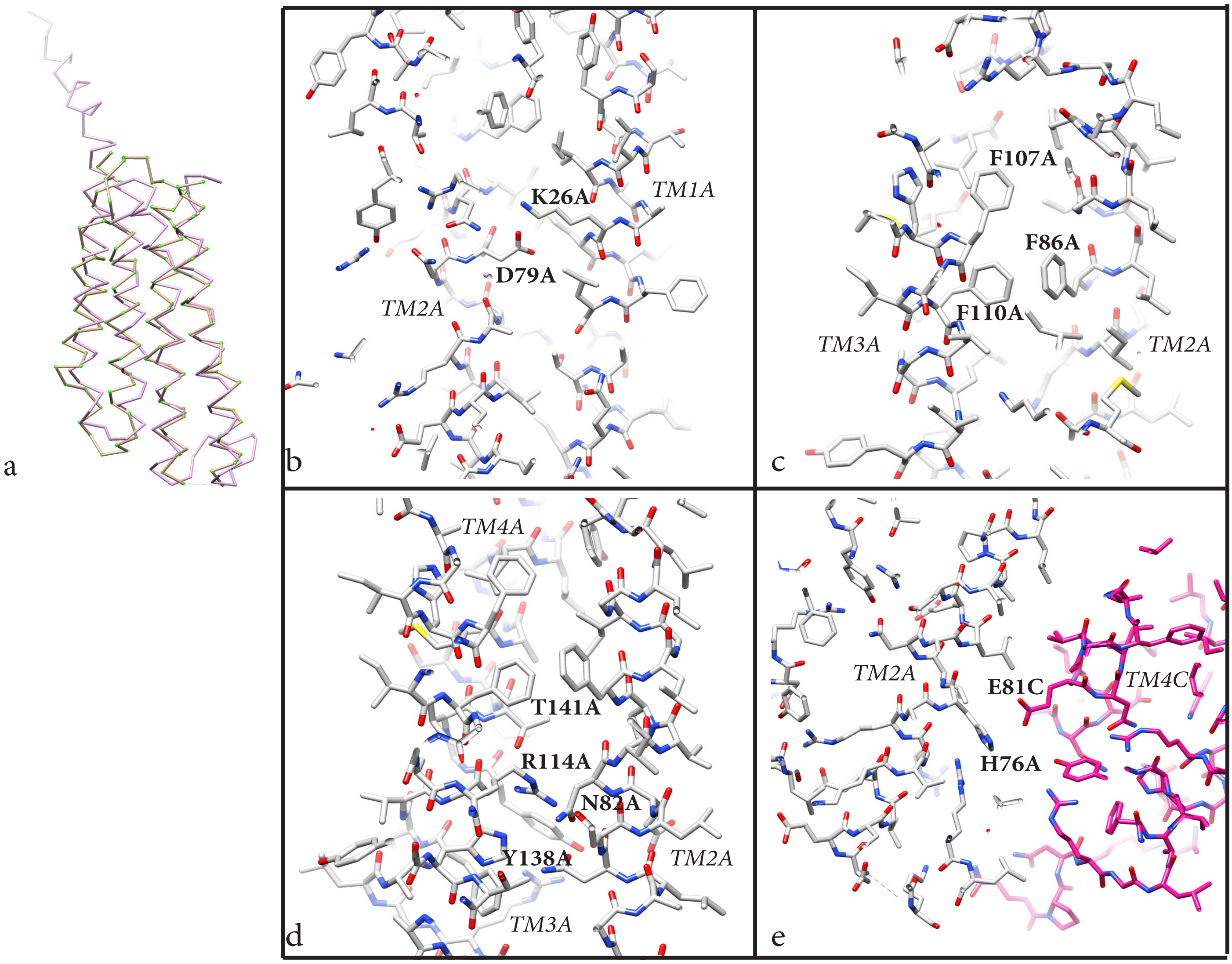

Supplementary Fig. S9 Helical packing of MGST1. (a) Comparison of the monomer of MGST1 to leukotriene C4 synthase (pdb 2uuh ref 11 in the main text). Cα atoms are shown (purple MGST1, yellow LTC4S). Root-mean-square deviations (rmsd in Å) after optimized fit of subsets of Cα atoms as determined by LSQAB in CCP4 are given in the text. (b) Intramolecular contact between K26 from TM1 and D79 from TM2. (c) Close intramolecular contacts between phenylalanine residues F86 from TM2 and F107, F110 from TM3. (d) Intramolecular contact network involving residues N82 from TM2, R114 from TM3 and Y138, T141 from TM4. (e) Intermolecular contact within the MGST1 trimer between H76 from TM2 and E81 from TM2 of a neighbouring monomer. A refers to subunit A (white) and C to subunit C (magenta).

Supplementary methods

2D crystallization and map generation

Recombinantly expressed and purified rat MGST1 formed large well-ordered 2D crystals using similar conditions as those previously found for protein purified from rat liver[1]. The specific activity from the present sample is 50-80 % as earlier reported (ref 47 in the main text). In preceding studies two co-existing crystal forms were observed[1]. For the present specimens there was a clear dominance of crystals with hexagonal p6 symmetry as compared to the orthorhombic form. Merging of electron diffraction data processed from untilted specimens showed that these p6 crystals were identical to those analyzed earlier both with regard to isomorphism having unit cell parameter a = b = 81.8 Å, and by comparison of amplitude data over the entire resolution range from 10 to 3 Å (data not shown). Thus, we concluded that it was justified to merge new electron diffraction data (Supplementary Figs. S5 and S6 online) with the previous set.

Electron diffraction patterns were recorded from specimens tilted up to 66.3° with an overall completeness of 82.5 % to a resolution of 3.0 Å in the plane of the membrane and 4.0 Å in the direction perpendicular to this plane (Supplementary Table S4, the native data set, online). Initial maps were calculated from the merged electron diffraction amplitudes combined with either previously determined experimental phases or using phases calculated from a poly-alanine version of the previous MGST1 model (ref 15 in the main text). These maps were comparable and could be used to build a matching first model. As an additional control, the structure of LTC4S (PDB ID: 2UUH) with all amino acid residues replaced by alanines was also used during initial modeling.

Although the resolution limit for the crystallographic refinement was similar to what was set in our previous work, 3.5 Å (ref 15 in the main text), the redundancy and completeness of the data was improved significantly leading also to better refinement statistics.

A difference map calculated between observed (with GSH) and model (without GSH) amplitudes and using model phases had the strongest density at a position facing the cytoplasmic side and at the interface between adjacent monomers of the MGST1 trimer. We interpreted this to arise from bound GSH molecules (Figs. 3a and b).

However, it should be noted that the present crystal form (p6) does not allow individual modelling of the subunits in the trimer as a symmetrisation averages the densities from GSH even if only one site of three would contain a GSH. It would have been interesting had it been possible and could have provided a result similar to the structure of diacylglycerol kinase[2] (PDB ID: 4UXX). This is also a homotrimer with a 1/3 of the sites reactivity and the ternary complex, crystallised in meso, showed only one of three sites occupied by an ATP analog.

MGST1-TNB map generation

Next, we recorded electron diffraction patterns from 2D crystals following soaking with TNB. 86 of these, from crystals tilted up to 57.4°, were of sufficiently good quality to be included in a 3D data set (supplementary Table S4, TNB data set, Supplementary Figs. S5 and S6 online). A difference map was calculated using the amplitudes from the two data sets and phases from the present MGST1 model.

References

1. Schmidt-Krey, I., Lundqvist, G., Morgenstern, R. & Hebert, H. Parameters for the two-dimensional crystallization of the membrane protein microsomal glutathione transferase. *J. Struct. Biol.* **123**, 87-96 (1989).

2. Li, D. *et al.* Ternary Structure Reveals Mechanism of a Membrane Diacylglycerol Kinase. *Nat. Comm.* **6**, 10140-10151 (2015).
